# A fast, low-cost, robust and high-throughput method for viral nucleic acid isolation based on NAxtra magnetic nanoparticles

**DOI:** 10.1038/s41598-023-38743-0

**Published:** 2023-07-20

**Authors:** Erlend Ravlo, Mirta Mittelstedt Leal de Sousa, Lise Lima Andersen, Mona Holberg-Petersen, Ingvild Klundby, Per Arne Aas, Lars Hagen, Sten Even Erlandsen, Janne Fossum Malmring, Zeeshan Ali, Anuvansh Sharma, Vegar Ottesen, Sulalit Bandyopadhyay, Magnar Bjørås

**Affiliations:** 1 Department of Clinical and Molecular Medicine (IKOM), Norwegian University of Science and Technology (NTNU), 7491 Trondheim, Norway; 2 Department of Microbiology, Oslo University Hospital and University of Oslo, Oslo, Norway; 3 Lybe Scientific, Erling Skjalgssons gate 1, 7030 Trondheim, Norway; 4 Department of Medical Microbiology, St. Olavs Hospital, 7006 Trondheim, Norway; 5 Particle Engineering Centre, Department of Chemical Engineering, Norwegian University of Science and Technology (NTNU), 7491 Trondheim, Norway

**Keywords:** Biotechnology, Infectious-disease diagnostics, Virology, Infectious diseases

## Abstract

The year of 2020 was profoundly marked by a global pandemic caused by a strain of coronavirus named severe acute respiratory syndrome coronavirus 2 (SARS-CoV-2), the etiological agent of coronavirus disease 2019 (COVID-19). To control disease spread, a key strategy adopted by many countries was the regular testing of individuals for infection. This led to the rapid development of diagnostic testing technologies. In Norway, within a week, our group developed a test kit to quickly isolate viral RNA and safely detect SARS-CoV-2 infection with sensitivity comparable to available kits. Herein, the procedure employed for the detection of SARS-CoV-2 in swab samples from patients using the NTNU-COVID-19 test kit is described in detail. This procedure, based on NAxtra magnetic nanoparticles and an optimized nucleic acid extraction procedure, is robust, reliable, and straightforward, providing high-quality nucleic acids within 14 min. The NAxtra protocol is adaptable and was further validated for extraction of DNA and RNA from other types of viruses. A comparison of the protocol on different liquid handling systems is also presented. Due to the simplicity and low cost of this method, implementation of this technology to diagnose virus infections on a clinical setting would benefit health care systems, promoting sustainability.

## Introduction

In December of 2019 a novel coronavirus, SARS-CoV-2, was identified in Wuhan, China^1,2^. The virus quickly spread throughout the world reaching nearly every country worldwide within 6 months, and the numbers of new cases and deaths rapidly increased^3,4^. On March 11, 2020, the World Health Organization declared the novel coronavirus outbreak a global pandemic^5^. As of January 31st 2023, over 753 million confirmed cases and 6.8 million global deaths were recorded in 195 countries according to the WHO Coronavirus Disease (COVID-19) Dashboard^6^.

Before vaccination programs were implemented worldwide, starting late 2020/early 2021^7,8^, regular testing of suspected cases along with other infection control measures (lockdown, contact tracing, quarantine, implementation of guidelines and information about risk factors and preventive measures to the public) were crucial to decrease the number of incidences and avoid deaths due to COVID-19^3^. At the initial stages of the pandemic, however, there was a massive shortage of personal protection equipment, general laboratory consumables and RNA extraction kits^9,10^. This heavily affected the testing capacity of many areas, which no longer could test potentially infected persons^11–14^. To overcome the shortage in diagnostic tests, decrease testing cost and reliance on commercial reagents, several labs designed in-house extraction protocols for RNA^12^. In Norway, our team developed the NTNU-COVID-19 diagnostic kit, in which NAxtra beads were used to extract nucleic acids from the corona virus. The method was approved for use in Norway and several million tests were delivered across the country and internationally, helping societies easing the burden of the pandemic^15^. The key for the NTNU-COVID19 kit success was the combination of an efficient, optimized virus lysis procedure and the isolation of virus nucleic acid using NAxtra beads, which are iron oxide paramagnetic nanoparticles (IONPs) coated with silica. Briefly, the extraction process is divided into 3 phases: a lysis- & binding phase, a washing phase, and an elution phase. The lysis phase, based on the protocols of Boom, R. et al.^16^ consists of mixing the sample (nasopharyngeal swab collected from patients) in an optimized guanidine thiocyanate (GITC)-based buffer, releasing the viral RNA into the solution, and stabilizing the RNA by inactivating RNAses potentially present in the mixture. In the binding phase, silica coated NAxtra magnetic nanoparticles suspended in isopropanol are added to the mixture, leading to direct interaction between the nucleic acids and the silica matrix. The paramagnetic properties of the nanoparticles enable their isolation along with bound RNA. In the washing phase, NAxtra magnetic nanoparticles are resuspended in different alcohols to remove proteins and cell debris. In the final step, nucleic acids are collected by suspending the NAxtra magnetic nanoparticles in a hypoosmotic elution buffer, following nucleic acid analysis via amplification of specific viral sequences by quantitative polymerase chain reaction (qPCR).

By using the NAxtra based method, we show that genetic material from both RNA and DNA viruses, non- enveloped or enveloped, can be successfully isolated and detected with high sensitivity, from as low as one particle of RNA or DNA per qPCR reaction. Furthermore, the procedure reported here is more user friendly than alternative methods, requiring less steps during sample preparation and eliminating steps where viscous solutions are employed. It is a low-cost method that requires shorter time, and a reduced amount of lab supplies to achieve similar results to commercially available kits. Furthermore, the procedure can be adapted for large scale screening using several liquid handling systems. Altogether, we show that the NAxtra method is faster, simpler, versatile for viral detection and flexible in terms of implementation on liquid handling systems compared to alternatives on the market. These features make the present procedure attractive for the standard diagnosis of viruses in the health care setting.

## Results

### Optimization of the NAxtra based method: input sample volume, nanoparticle concentration and overall test time

For standard detection of SARS-CoV-2 in a clinical setting, a nasopharyngeal swab is collected and transferred to a vial containing a small volume **(~ **1 mL**)** of viral transport medium (VTM). This sample is then transported to a laboratory for testing, where chemicals or heating are employed to lyse and inactivate viruses. A fraction of the swab sample is further used for isolation of the viral nuclei acid material, either by using RNA-purification columns or magnetic beads. The eluate, consisting of purified RNA, is then amplified, and analyzed via qPCR using primers targeting specific regions of the viral genome.

Our first goal was to determine the ideal volume of input sample to accurately detect SARS-CoV-2 in patient samples using the NAxtra method. In the standard procedure, 100 µL of the swab sample (input) are mixed with lysis buffer (200 µL) prior to incubation with 130 μg of NAxtra nanoparticles previously suspended in 400 μL isopropanol. We found that the detection of SARS-CoV-2 was improved by doubling the input volume (200 µL) and increasing the isopropanol volume to 600 µL (Supplementary Fig. 1). Different dilutions of the initial input volumes were also tested confirming an improvement in virus detection for the doubly input sample at all dilutions compared to the standard input amount. Accurate SARS-CoV-2 detection was observed for both standard and doubly input samples diluted down to 1:1000 (Supplementary Fig. 1A) and improvement in detection by doubling the input volume was also observed upon further dilution to as low as 1:8000 (Supplementary Fig. 1B). As the increased amount of input sample improved viral detection, the following assays were performed using 200 uL input samples and 600 uL isopropanol.

To assess whether an increased concentration of the nanoparticles would increase the sensitivity of the test, a doubling of the nanoparticle concentration was tested (260 μg per reaction, 0.43 mg/ml of nanoparticles in isopropanol) for SARS-CoV-2 RNA isolation. According to Supplementary Fig. 2, an increase in nanoparticle concentration did not lead to higher sensitivity and did not improve the detection of SARS-CoV-2 at the lowest concentration. Therefore, it is likely that the NAxtra nanoparticles were not yet saturated with nucleic acids at a concentration of 130 μg per reaction. Thus, this concentration was selected as default in the following experiments.

The earliest NAxtra protocol developed for the KingFisher Flex automated system used a total of 44 min for extraction, with much of the time spent on washing steps and a drying step prior to elution. To increase throughput, the method was further optimized to maintain sensitivity while reducing the extraction time (Supplementary Fig. 3). The faster protocol requires 14 min from plate insertion into the KingFisher Flex system until completed extraction. This is a drastic reduction in workflow time, with only minimal differences in sensitivity (Supplementary Fig. 3A). Further serial dilution to 1:8000 indicated similar performance (non-significant difference) when comparing the standard and faster procedures in most cases (Supplementary Fig. 3B). Due to the advantage of shorter diagnostic time without drastic losses in sensitivity, further experiments were performed using the 14 min protocol.

### Comparison between NAxtra and MagMAX viral/pathogen II (MVP II)

The sensitivity of the method was tested using the Amplirun total SARS-COV-2 control kit (Vircell), which includes a quantified number of viruses in each provided sample. For comparison, the extraction was performed in parallel with a commonly used viral RNA extraction kit (MagMAX viral/pathogen II (MVP II)) available on the market. The samples from Vircell were subsequently diluted so that the sensitivity for both extraction kits could be compared at lower titers.

**Figure 1:**
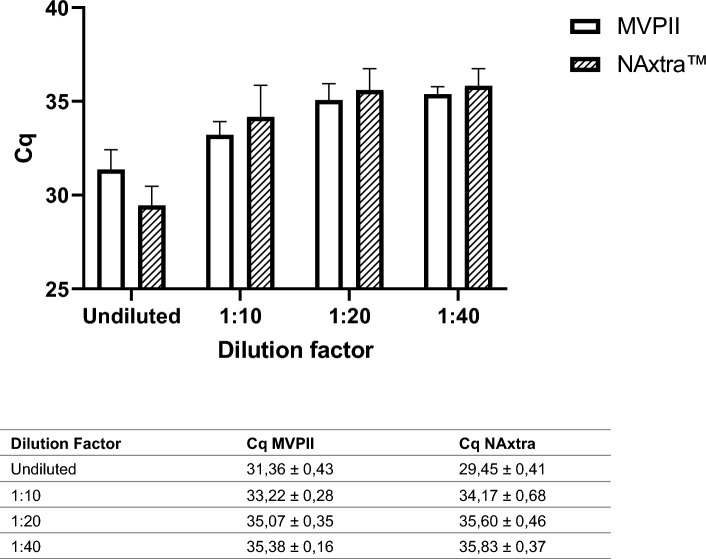
Comparison with another nucleic acid extraction product on the market (MagMAX viral/pathogen II (MVP II)), using a dilution series made from Vircell SARS-CoV-2 panel. Error bars show the 95% CI for 3 independent NA extractions. Cq and corresponding error bar values are also provided in the associated table.

The NAxtra protocol performs on par with the MVPII extraction method (Fig. 1). The MVPII protocol utilizes a heating step which gives it an extraction runtime of approximately 22 min, as opposed to the 14 min of the NAxtra protocol. This heating step is likely included to increase the efficiency of the protease K, added as part of the MVPII protocol.

### Adaptation of the NAxtra method in automated liquid handlers

The NAxtra method was designed to be flexible, easily adaptable to new platforms. Thus, the robustness and reliability of the method was tested by comparing its efficiency to commonly used nucleic acid extraction methods/kits in different automated liquid handling platforms.

**Figure 2:**
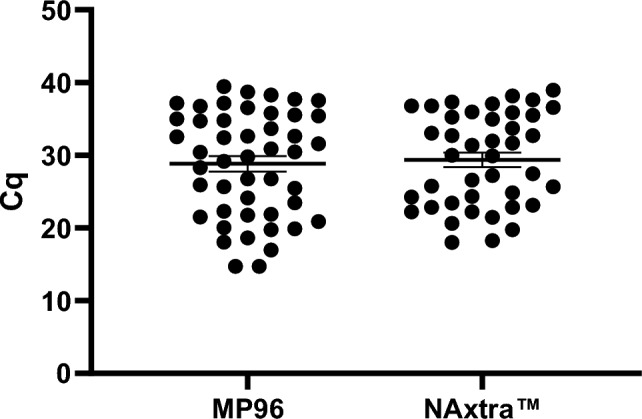
Validation data of the NAxtra protocol on a Tecan Fluent 1080 robot platform. 47 samples from COVID-19 patients were used to evaluate nucleic acid extraction using the MagNA Pure 96 (MP96) and NAxtra methods. Eluates were analyzed using commercially available PCR reagents (Superscript). Error bars represent standard error of the mean (SEM).

The implementation of the NAxtra method on the Tecan platform was performed in parallel with nuclei acid extractions using the MagNA Pure 96 (MP96) kit (Roche). The eluates after extraction using the NAxtra and MP96 procedures were tested with the same PCR mixtures. As illustrated in Fig. 2, the NAxtra method is compatible with the Tecan Fluent platform, displaying equivalent sensitivity to the MP96. Nucleic acid extraction with the MP96 method was performed with double the input-volume as compared to NAxtra.

**Figure 3:**
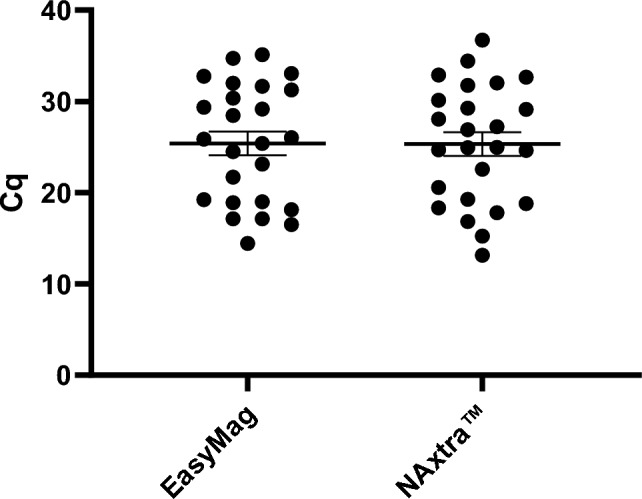
Validation data of the NAxtra protocol on the KingFisher Flex system. Tested on 25 different COVID-19 patient samples. Error bars in SEM.

The validation of the NAxtra method on the KingFisher was also performed in parallel with extractions using the EasyMag protocol. According to Fig. 3, the two extraction methods show comparable results. Altogether, the data presented in Figs. 2 and 3 show that the method is easily tailored to different robotic liquid handling systems. The NAxtra method has also been tested and implemented on Hamilton Microlab STAR and KingFisher Duo.

### Compatibility with downstream PCR

**Figure 4:**
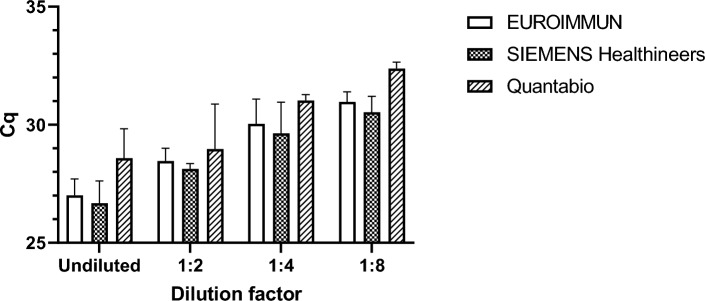
qPCR reagent comparisons from three vendors using RNA-containing eluates extracted from a SARS-CoV-2 patient sample. The nucleic acid extraction was performed using the NAxtra method. Error bars show the 95% CI for 3 independent NA extractions for each dilution.

To determine the compatibility of the method with downstream PCR, nucleic acids extracted using the NAxtra protocol were tested with the following PCR kits: EURORealTime SARS-CoV-2 (EUROIMMUN), FastTrack FTD SARS-CoV-2 assay (SIEMENS Healthineers), and qScript XLT 1-Step RT-qPCR ToughMix (Quantabio) (Fig. 4).

Detection of SARS-CoV-2 with the three PCR tested kits was successful, displayed somewhat similar performance. Although not statistically significant, there was a trend of higher sensitivity (lower Cq values) when detecting SARS-CoV-2, at all tested dilutions, with the EUROIMMUN and the SIEMENS Healthineers kits. Nevertheless, this data confirms that purification of SARS-CoV-2 RNA via the NAxtra method is suitable for downstream SARS-CoV-2 quantification using different PCR kits.

### Sensitivity of NAxtra method on respiratory pathogens

**Figure 5:**
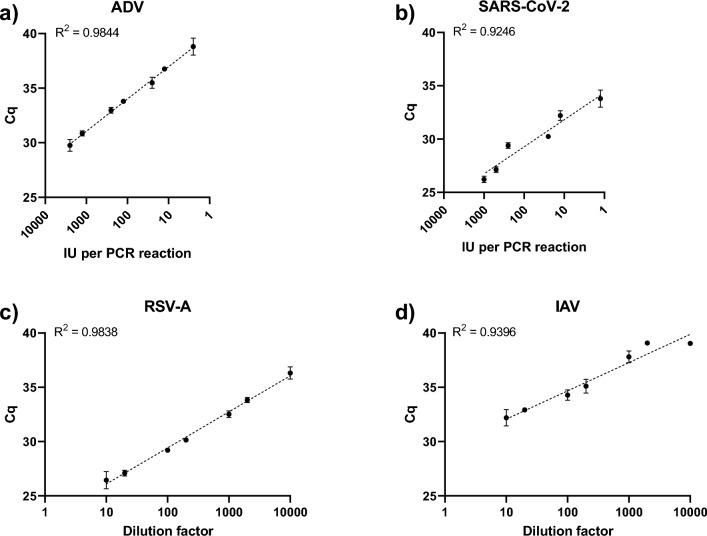
Application of the NAxtra method on four Qnostics analytical panels. Panels (**a**) and (**b**) had quantified number of viruses, while prearranged dilutions were provided for (**c**) and (**d**). Eluates were analyzed via qPCR. Error bars show the standard deviation from 3 extractions for ADV, IAV & SARS-CoV-2 and 4 independent NA extractions for RSV-A at each dilution.

The NAxtra extraction method was further tested on four pathogen panels: influenza A (IAV, ref: INFAAQP), respiratory syncytial virus A (RSV A, ref: RSVAAQP), adenovirus (ADV, ref: ADVAQP), and SARS-CoV-2 (ref: SCV2AQP) (Fig. 5). These Qnostics panels allowed for the sensitivity of the method to be tested, not only for detection of SARS-CoV-2, but for a wider range of respiratory viruses.

The NAxtra method proved capable of nucleic acid extraction from non-enveloped double-stranded DNA viruses (Fig. 5a) and enveloped single-stranded RNA viruses (Figs. 5b–d). Notably, data on ADV and SARS-CoV-2 show that the NAxtra method can detect as low as one virus per PCR reaction (Figs. 5a,b). Although the RSV and IAV panels did not include a quantified number of viruses, the NAxtra method enable the detection of these viruses at a 10,000-dilution factor (Figs. 5c,d). The detection rates for dilutions are provided in Supplementary Table 2. Based on these results, the NAxtra method can be considered a versatile and fast diagnostic tool in nucleic acid-based detection assays.

## Discussion

Here, we report an RNA/DNA extraction method based on the NAxtra magnetic nanobead technology. We tested the sensitivity and selectivity of this method in purifying nucleic acids compared to leading products on the market and tested its compatibility with downstream qPCR kits. The NAxtra method for nucleic acid extraction was proven to be as sensitive as commercially available alternatives, allowing nuclei acid isolation within 14 min and extraction of very low numbers of virus particles for downstream detection via PCR. Moreover, the method allows successful isolation of RNA or DNA from non-enveloped or enveloped viruses, and the procedure can be easily adapted for large-scale testing using automated liquid handling platforms. Due to the simplicity, versatility, fast read-outs, throughput, low-cost and high sensitivity, the NAxtra method is an attractive option for clinical diagnosis of infections caused by different types of viruses.

## Methods

### Cohort and materials

The diagnostics of SARS-CoV-2 for the majority of community and hospital samples from Oslo and parts of south-eastern Norway is conducted at the Department of Microbiology at OUH^17^. These samples consist of throat/nasopharyngeal swab specimens collected from individuals with suspected infection. For diagnostic purposes, a fraction of each swab sample was used for primary detection of SARS-CoV-2 positive samples using the protocol originally developed at NTNU^15^ operating on an automated Tecan Fluent 1080 workstation (Tecan Trading AG, Switzerland). The NAxtra beads and NAxtra lysis buffer employed in the NTNU protocol are currently commercially available from Lybe Scientific. The remainders of the swab samples were used by our research group to further optimize the standard nucleic acid extraction procedure using NAxtra beads and lysis buffer (Lybe Scientific) and to validate the compatibility of the method with distinct downstream PCR procedures, its sensitivity in detecting a range of viruses and adaptability to several automated platforms for high-throughput measurements.

### Nucleic acid isolation

#### NAxtra on KingFisher flex

Inputs of 200 µL were mixed with 200 µL NAxtra LYSIS BUFER (Lybe Scientific). To each sample, it was added a 600 µL mixture of 20 µL NAxtra MAGNETIC BEADS (Lybe Scientific) in 580 µL isopropanol. Lysis, binding, washing, and elution were performed on the KingFisher Flex Purification System with a 96 Deep-Well Head (Thermo Scientific). Elution was generally performed in 50 µL of nuclease free water. When comparing to the MagMAX viral/pathogen II (MVP II) Nucleic Acid Isolation Kit (Applied Biosystems), elution volumes were 100 µL (Fig. 1).

#### MagMAX viral/pathogen II (MVP II) nucleic acid isolation kit

The MagMAX viral/pathogen II (MVP II) nucleic acid isolation kit (Applied Biosystems) is a nucleic acid purification kit based on magnetic bead technology on the market. Extraction of viral nucleic acid was conducted on the KingFisher Flex Magnetic Particle Processor with a 96 Deep-Well Head (Thermo Scientific) according to kit instructions. Sample volumes were 200 µL, with elution volumes of 100 µL.

#### MagNA pure 96 (MP96) and EasyMag

Viral nuclei acid extraction using the MagNA Pure 96 (MP96) (Roche) and EasyMag (ABP Biosciences) kits were performed according to manufacturers’ instructions.

### Viral detection via real-time RT-PCR

#### Panels for virus quantification

To test the sensitivity of the NAxtra method at low titers, the Amplirun total SARS-COV-2 control panel (Vircell, MBTC030) was used as input sample (resuspended at 42 copies/µL) in Fig. 4. The Qnostic panels ADV Analytical Q Panel (ADVAQP), RSV-A Analytical Q Panel 01 (RSVAAQP01-B), IAV Analytical Q Panel 01 (INFAAQP01-B), SARS-CoV-2 Analytical Q Panel (SCV2AQP) were used in Fig. 5. The analytical panels consist of prearranged viral solutions, where exact viral concentrations were provided for ADV and SARS-CoV-2. Viral concentrations and dilutions are available at Qnostics websites. For each dilution, three (or four for RSV-A) independent nucleic extractions were performed on a KingFisher Flex system (Thermo Fisher Scientific). For the adenovirus PCR, each reaction consisted of 12.5 µl Quantabio qScript XLT One-Step RT-qPCR ToughMix, with a 0.5 µL of an adenovirus A primer/probe mix, 5 µl of eluate and 7 µl nuclease free water. For respiratory syncytial virus A PCR, each reaction contained 12.5 µl Quantabio qScript XLT One-Step RT-qPCR ToughMix, with a 0.475 µL of RSV A primer/probe mix, 5 µl of eluate and 7.025 µl nuclease free water. For influenza A PCR, 10 µl Quantabio qScript XLT One-Step RT-qPCR ToughMix, with a 0.32 µL of RSV A primer/probe mix, 5 µl of eluate and 4.68 µl nuclease free water were used per reaction. R-squared values, calculated based on nonlinear regressions for semilog lines, were above 0.92 indicating that the extraction method is of high precision.

#### Real-time RT-PCR

Eluates from nucleic acid extraction (2.5 µL of the total eluate volume, 50 µL) were analyzed by real-time RT-PCR using a CFX96 Touch Real-Time PCR Detection System (Bio-Rad) in a reaction volume of 10 µl containing Quantabio qScript XLT One-Step RT-qPCR ToughMix and specific primers for SARS-CoV-2 detection (Supplementary Table 1). In the validation of nucleic acid eluates using downstream PCR kits available on the market, illustrated in Fig. 4, qPCR was performed according to manufacturers’ instructions (EURORealTime SARS-CoV-2 (EUROIMMUN, MP 2606-0100), FastTrack FTD SARS-CoV-2 assay (SIEMENS Healthneers, FTD-114-96), and qScript XLT 1-Step RT-qPCR ToughMix (Quantabio, ref: QUNT95132-02 K)). For the detection of SARS-CoV-2 RNA, thermal cycling was performed at 50 °C for 10 min followed by 95 °C for 1 min and then 45 cycles of 95 °C for 3 s and 60 °C for 30 s. For the detection of RSV-A, thermal cycling was performed at 50 °C for 15 min followed by 95 °C for 2 min and then 45 cycles of 95 °C for 15 s and 60 °C for 30 s. Similar protocol was employed for the detection of IAV, except for the first step, where thermal cycling was performed for 10 min instead of 15 min. Detection of Adenovirus was conducted at 95 °C for 10 min, followed by 45 cycles of 95 °C for 15 s and 60 °C for 60 s. The primers for RSV-A, IAV, and adenovirus were synthesized by Integrated DNA Technologies (Leuven, Belgium), while the SARS-CoV-2 primers/oligonucleotides were purchased from Sigma-Aldrich. A list of primers used in his study is provided in Supplementary Table 1. All probes used for these qPCRs were TaqMan-probes.

### Ethics declarations

The nasopharyngeal swab samples used in this study were collected in the context of routine clinical patient care and the assays were performed on residual de-identified patient material in a Norwegian-accredited diagnostic service laboratory supporting diagnostic processes including RNA extractions for the standard RT-PCR assay; thereby not requiring informed consent and ethics committee approval, in compliance with IRB regulations.

## Supplementary Information

Supplementary Information.

## Data Availability

The authors confirm that the data supporting the findings of this study are available within the article and its supplementary materials.
