# Supplementary material for: A fast, low-cost, robust and high-throughput method for viral nucleic acid isolation based on NAxtra magnetic nanoparticles
Source: Sci Rep. 2023 Jul 20;13:11714. doi: 10.1038/s41598-023-38743-0 (PMC10359305; doi:10.1038/s41598-023-38743-0)
Supplement: Supplementary file 1 — Supplementary Information. [file 41598_2023_38743_MOESM1_ESM.pdf]

A fast, low-cost, robust and high-throughput method for viral nucleic acid isolation based on NAxtra™ magnetic nanoparticles

Supplementary table 1. Primers used for viral detection via qPCR.

Virus	Primer Sequence (5′ to 3′)	Acc. Nr.	Target Gene	Source
RSV-A	(+)-ATGGCTCTTAGCAAAGTCAA	M11486	N gene	[18] *
	(−)-GCTCCGTTGGATGGTGTA			
	[Cy5]ACACTCAAC /TAO/ AAGATCAACTTCTGTCATC[IBRQ][a)]			
INF-A	(+)-AAGACCAATYCTGTCACCTCTGA	D10598	Matrix protein (M1)	[19] *
	(−)-CAAAKCGTCTACGCTGCAGTCC			
	[6-FAM]TTTGTRTTY /ZEN/ ACGCTCACCGTGCC[IBFQ] [b)]			
Adenovirus A, B, D	(+)-TCCGTCAAGAGGCCACTC	X73487 AY601636 AF099665	5' non-coding region	IH[e)]
	(+)-CCCGTCAAGAGACTACTC			
	(−)-CGGAGCTCAGAGAAATCTC			
	(−)-GCGGAGGAGAAAACTCT			
	(−)-AGCAAAGTTTAGAGAAAACTCT			
	(−)-GCTGGCAGAGAAAACTCT			
	[6-FAM]ct+Cg+Ctgg+Ca+Ct+Caa[IBFQ][c)]			
SARS-CoV-2	(+)-ACAGGTACGTTAATAGTTAATAGCGT	NC_004718	E gene	[20]
	(−)-ATATTGCAGCAGTACGCACACA			
	[6-FAM]-ACACTAGCCATCCTTACTGCGCTTCG[BHQ1] [d)]			

(+) Forward primers; (−) reverse primers; Acc.Nr: GeneBank accession number; Reporter and double-quencher dyes used for probe labeling: [a)] 5′ Cy5/TAO/3′ Iowa Black RQ (5'Cy5/TAO/3'IBRQ), [b,c)] 5′ 6-carboxyfluorescein/ZEN/3′ Iowa Black FQ (5′6-FAM/ZEN/3′IBFQ), and [d)] 6-FAM/ Black Hole Quencher 1 (BHQ1). *Minor modifications were introduced on original sequences. [e)] Sequences designed in-house.

Supplementary table 2. Detection rates of NAxtra eluates via qPCR using four Qnostics analytical panels.

Virus	IU per PCR reaction[1] or Dilution Factor[2]	Cq Values	SD	No. of positive results/ no. of tested samples
ADV[1]	2500	29.75	0.55	3/3
	1250	30.86	0.24	3/3
	250	32.97	0.27	3/3
	125	33.79	0.22	3/3
	25	35.49	0.50	3/3
	12.5	36.75	0.21	3/3
	2.5	38.81	0.77	2/3
SARS-CoV-2[1]	1000	26.22	0.3	3/3
	500	27.13	0.26	3/3
	250	29.39	0.26	3/3
	25	30.24	0.1	3/3
	12.5	32.21	0.46	3/3
	1.25	33.8	0.81	3/3
RSV-A[2]	10	26.44	0.79	4/4
	20	27.09	0.26	4/4
	100	29.20	0.21	4/4
	200	30.15	0.12	4/4
	1000	32.51	0.31	4/4
	2000	33.84	0.24	4/4
	10000	36.32	0.57	4/4
IAV[2]	10	32.20	0.75	3/3
	20	32.92	0.11	3/3
	100	34.28	0.47	3/3
	200	35.11	0.64	2/3
	1000	37.81	0.54	2/3
	2000	39.08	NA	1/3
	10000	39.05	NA	1/3
	20000	NA	NA	0/3

[1]IU (International Unit) per PCR reaction values were calculated based on exact viral number per dilution provided in the Qnostic panels for ADV and SARS-CoV-2. [2]Only dilution factors are available for RSV-A and IAV Qnostic panels. SD. Standard deviation. No: Number.

Supplementary Figure 1.

A

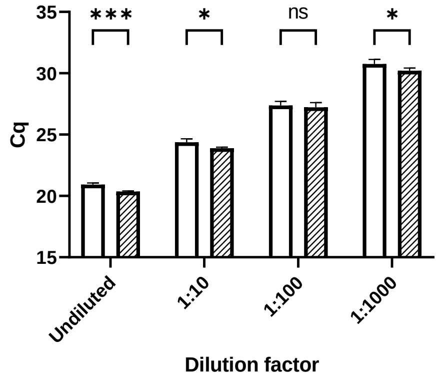

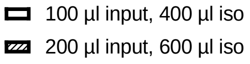

Dilution Factor	Cq 100 µl input, 400 µl iso	Cq 200 µl input, 600 µl iso
Undiluted	20,92 ± 0,15	20,36 ± 0,05
1:10	24,37 ± 0,29	23,90 ± 0,08
1:100	27,37 ± 0,35	27,22 ± 0,38
1:1000	30.76 ± 0,37	30,22 ± 0,21

B

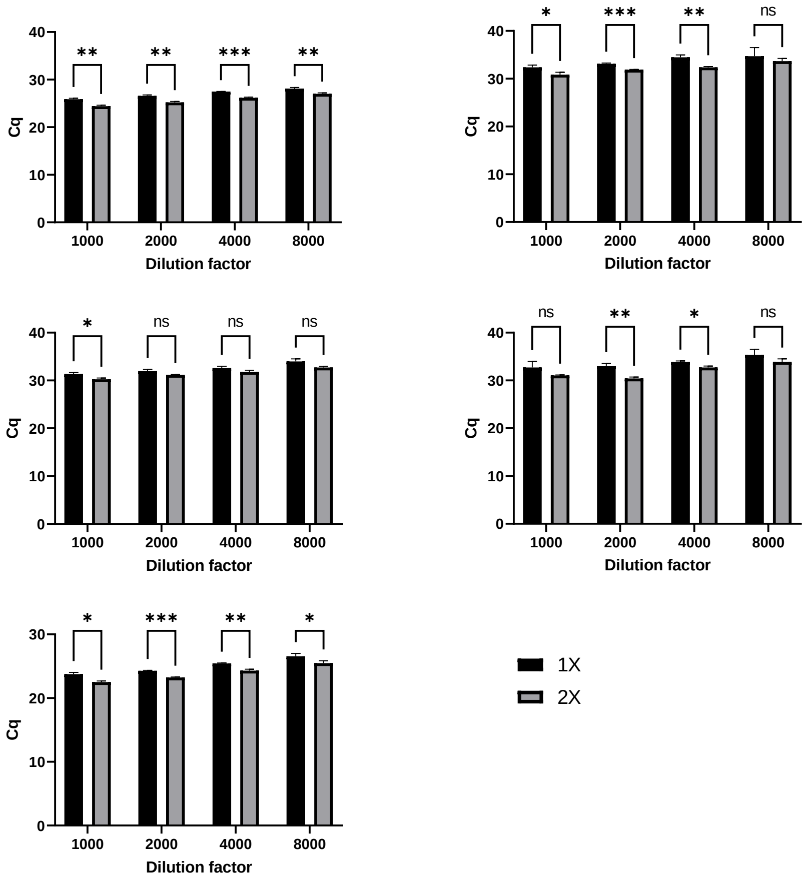

Supplementary figure 1. Cycle number past threshold (Cq) for SARS-CoV-2 extractions from swab samples from COVID-19 patients using standard (100 µL) or double (200 µL) sample input volume. (A) data from one patient, dilutions up to 1:1000. Cq and corresponding error bar values are provided in the associated table. (B) Additional data from 5 patients, dilutions ranging from 1:1000 to 1:8000. 1X: 100 µL sample input volume; 2X: 200 µL sample input volume. In all cases, RNA isolation was performed using 130 µg NAxtra^{TM} beads suspended in 400 µL (beads concentration of 0.33 mg/mL) or 600 µL (beads concentration of 0.33 mg/mL) isopropanol, respectively. Error bars represent standard deviation of 6 independent NA extractions for each dilution. (***) were highly significant when compared with 100 µL input measurements, p-value ≤ 0.001. (**) indicates p-value ≤ 0.01, (*) indicates p-value ≤ 0.05 and non-significant (ns) was assigned to p-value > 0.05 with the Student t-test.

Supplementary Figure 2.

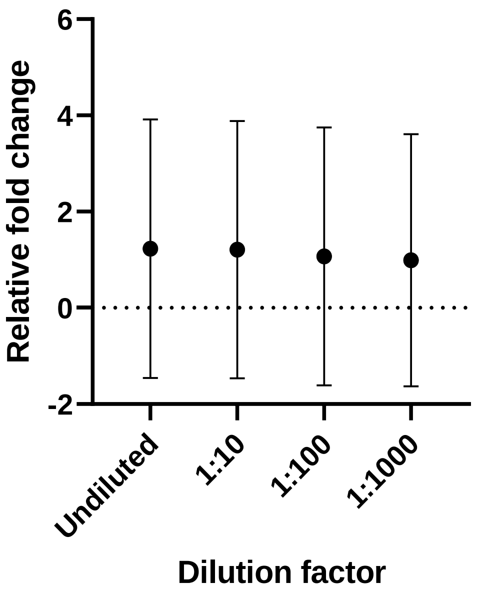

Supplementary figure 2: Relative fold change detection of SARS-CoV-2 RNA extractions by qPCR using different concentrations of NAxtra™ nanoparticles. Values for undiluted (260 ug beads relative to the default, 130 ug beads) and diluted beads are indicated. SARS-CoV2 RNA isolations were performed using 200 µL input samples and NAxtra™ beads suspended in 600 µL isopropanol. Error bars show the 95% confidence interval (CI) for 3 independent NA extractions for each dilution.

Supplementary Figure 3.

A

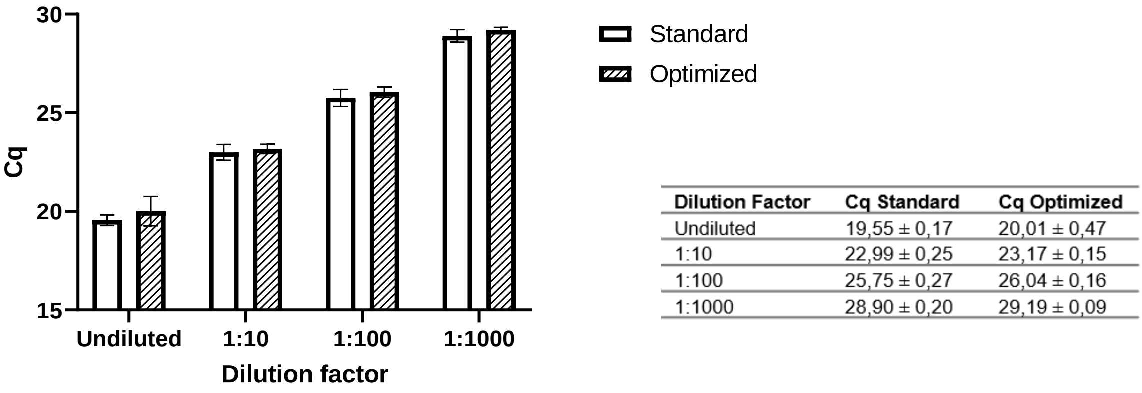

Dilution Factor	Cq Standard	Cq Optimized
Undiluted	19,55 ± 0,17	20,01 ± 0,47
1:10	22,99 ± 0,25	23,17 ± 0,15
1:100	25,75 ± 0,27	26,04 ± 0,16
1:1000	28,90 ± 0,20	29,19 ± 0,09

B

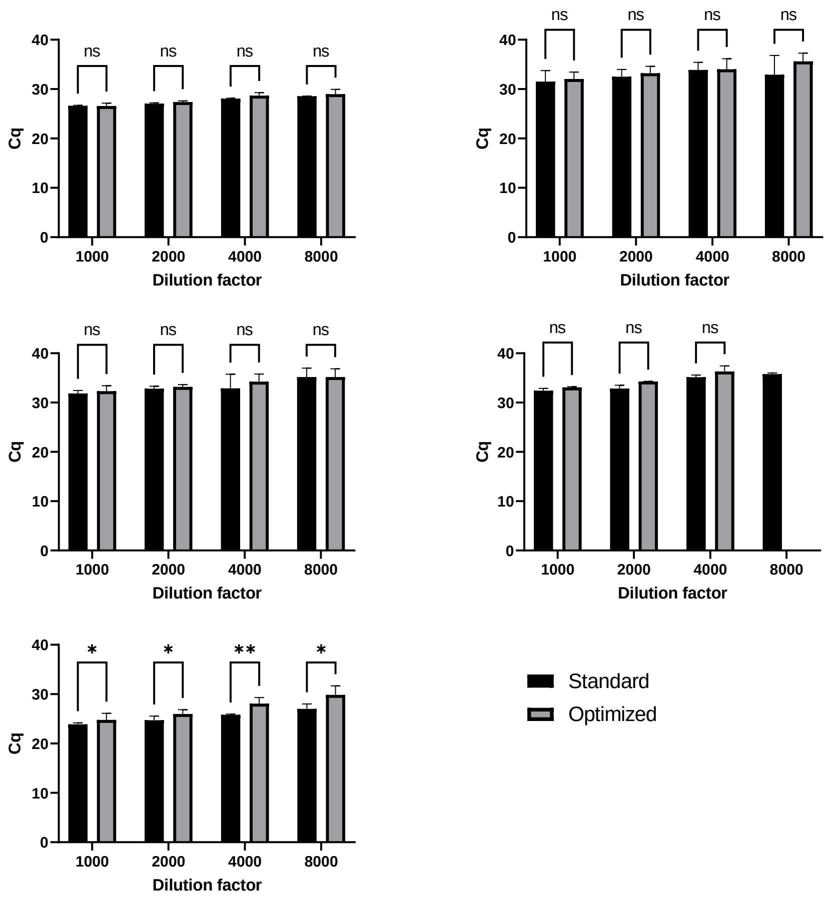

Supplementary figure 3: Cycle number past threshold (Cq) for SARS-CoV-2 extractions using the 44 minutes standard protocol vs the 14 minutes optimized protocol. (A) Data from a single patient, sample dilutions up to 1:1000 factor. Cq and corresponding error bar values are also provided in the associated table. (B) Additional data from 5 patients, dilutions ranging from 1:1000 to 1:8000. Student t-test revealed non-significant differences between most measurements obtained using standard and optimized protocols at indicated dilutions in A and B, supporting that reduction in turnaround time does not critically affect the quality of the measurements. Error bars show the 95% CI for 4 independent NA extractions for each dilution. p-value ≤ 0.001. (**) indicates p-value ≤ 0.01, (*) indicates p-value ≤ 0.05 and non-significant (ns) was assigned to p-value > 0.05 with the Student t-test.
